# Adenosine A2a Receptor Stimulation Mitigates Periodontitis and Is Mitoprotective in Gingival Fibroblasts Promoting Cellular Resilience

**DOI:** 10.3390/cells14161266

**Published:** 2025-08-16

**Authors:** A. C. Morandini, S. Dawson, N. Paladines, N. Adams, E. S. Ramos-Junior

**Affiliations:** 1 Department of Oral Biology, Dental College of Georgia, Augusta University, Augusta, GA 30912, USAeramosjunior@augusta.edu (E.S.R.-J.); 2 Department of Periodontics, Dental College of Georgia, Augusta University, Augusta, GA 30912, USA

**Keywords:** periodontitis, fibroblast, mitochondria, A2aR, adenosine receptor

## Abstract

Adenosine signaling plays protective roles in gingival mitochondrial health and inflammation control, with the ectoenzyme CD73 implicated in periodontitis. Here, we investigated the effects of selective adenosine A2a receptor (A2aR) stimulation using the agonist CGS21680 in a mouse model of ligature-induced periodontitis (LIP) and in gingival fibroblast mitochondrial function. Mature C57Bl/6 mice underwent LIP and received daily intraperitoneal injections of CGS21680 (0.1 mg/Kg) or saline. After 8 days, gingival tissues and maxillae were analyzed for alveolar bone loss and Il-1β levels. In parallel, murine gingival fibroblasts (mGFs) were treated with Tnf-α (5 ng/mL) ± CGS21680 (10 µM) to assess mitochondrial function, morphology, and quality control. A2aR activation significantly reduced alveolar bone loss and Il-1β expression in vivo. In vitro, CGS21680 suppressed Tnf-α-induced Cxcl10 and Cxcl12 expressions and enhanced Vegf production. Mitochondrial analysis revealed increased mitochondrial complex levels, membrane potential, and mass, alongside reduced reactive oxygen species (ROS), proton leak, and mitochondrial stress. Ultrastructural studies showed elongated, healthier mitochondria and increased pro-fusion markers, indicating enhanced mitochondrial quality control. Overall, A2aR stimulation attenuates periodontal inflammation and confers mitoprotective effects on gingival fibroblasts, supporting its potential as a therapeutic strategy to both mitigate periodontitis progression and preserve tissue bioenergetics supporting cellular resilience.

## 1. Introduction

Periodontitis is a chronic inflammatory disease characterized by the interplay among microbial challenge [1,2], host immune response [3,4], and tissue metabolism [5,6], driven by dysregulated immune responses [7] and metabolic dysfunction in stromal cells, like gingival fibroblasts [8]. Extracellular adenosine, produced by the ectoenzymes CD39/CD73, has been shown to attenuate inflammation in IL-1β-stimulated gingival fibroblasts via stress-regulating enzymes, such as heme-oxygenase 1 (HO-1), and metabolic sensors, such as adenosine monophosphate-activated protein kinase (AMPK) [9], and to protect against alveolar bone resorption through the modulation of osteoclast activity [10]. The ectonucleotidases CD39 and CD73 are the two major cell-surface-associated enzymes, which function to generate adenosine from extracellular adenosine triphosphate [11]. While the anti-inflammatory [12] and bone-protective roles [13] of adenosine have been documented, the specific adenosine receptor mediating these effects within the periodontium have remained unclear.

The adenosine A_2_a receptor (A_2_aR) is a G-protein-coupled receptor known to suppress inflammatory signaling [14], being recognized as an essential part of the physiological negative feedback mechanism for the limitation and termination of both tissue-specific and systemic inflammatory responses [15]. Logically following our previous studies and based on evidence that CGS21680, a selective A_2_aR agonist, can mitigate inflammation, preventing liver damage [15] and lung inflammation with fibrosis [16] and being mitoprotective in an osteoarthritis model [17], we hypothesized that A_2_aR stimulation underpins adenosine’s protective effects in periodontal disease and could improve mitochondrial health in gingival fibroblasts, ultimately promoting cellular resilience. To test whether adenosinergic signaling via A2aR would dampen periodontal inflammation, protecting the alveolar bone and conferring mitoprotective effects in gingival fibroblasts, we employed both an in vivo ligature-induced periodontitis model, administering CGS21680 throughout the disease course, and in vitro assays using Tnf-α-stimulated murine gingival fibroblasts pretreated with the agonist to dissect receptor-specific mechanisms underlying inflammation and bone loss prevention. We further investigated the mitochondrial dynamics by which A_2_aR signaling could confer cellular resilience [18].

Given previous findings linking extracellular adenosine to mitochondrial biogenesis and metabolic reprogramming in gingival fibroblasts [8], we conducted assays measuring electron transport chain protein expression, mitochondrial membrane potential and mass, oxygen consumption rate, ultrastructural morphology, and fusion/fission dynamics to ultimately access mitochondrial health status [19]. This multifaceted strategy allowed us to determine whether A_2_aR activation not only dampens inflammation and bone loss in vivo but also preserves mitochondrial quality through enhanced fusion-driven dynamics—offering a cohesive understanding of the roles of adenosine in periodontitis mitigation and in cellular resilience.

## 2. Methods

### 2.1. Animal Ligature-Induced Periodontitis (LIP) and Adenosine A2aR Treatment in Vivo

The experimental periodontitis model was conducted as previously described [10], using adult C57BL/6 mice (3–6 months) males and females bred in-house (*n* = 5/group). Briefly, a 5–0 silk ligature was placed around the upper right second molar, while the left upper second molar was left unligated as an internal control. An adenosine A2aR agonist (CGS21680; Tocris, Minneapolis, MN, USA, #1063/10) or a saline control was injected daily at a dose of 0.1 mg/Kg intraperitoneally, starting one day before the ligature placement. Animals were euthanized by carbon dioxide followed by cervical dislocation as a secondary method after 8 days of LIP, and hemimaxillae were harvested for assessment of alveolar bone loss by microcomputed tomography (micro-CT). Gingival tissues were processed for mRNA and protein analysis, and primary murine gingival fibroblasts were primarily cultured for further experimental analysis.

### 2.2. Primary Murine Fibroblast Cell Culture and Treatments

Primary murine gingival fibroblasts (mGFs) were isolated and cultured as previously described [10]. For cell treatments, 5 ng/mL mouse recombinant Tnf-α (R&D Systems, Minneapolis, MN, USA, #410-MT) and 10 µM CGS21680 (Tocris, #1063/10) were used for the time indicated in each figure legend.

### 2.3. RNA Isolation, cDNA Synthesis, and Quantitative PCR (RT-qPCR)

The total RNA was extracted from 1 × 10^5^ cells/well, using the Invitrogen PureLink RNA Mini Kit (ThermoFisher Scientific, Waltham, MA, USA), and reverse transcription was performed with SuperScript IV VILO Master Mix (ThermoFisher Scientific), as previously described [8,10]. The following inventoried Taqman assays for quantitative PCR were used: murine *Mfn1* (Mm00612599_m1), *Mfn2* (Mm00500120_m1), and *Fis1* (Mm00481580_m) in a 10 µL final volume with TaqMan Fast Advanced Master Mix in a StepOne Plus Real-Time PCR system (Life Technologies, Carlsbad, CA, USA). Relative quantitation was performed using the reference gene assay *Actb*-Mm02619580_g1 in duplex reactions using the comparative Ct (∆∆Ct) method. Analysis was performed using StepOne Plus software v.2.3 and Graph Pad Prism v.10.

### 2.4. ELISA

Protein levels of inflammatory cytokines Cxcl10, Cxcl12, and Vegf were measured in cell supernatants of mGFs after 24 h of Tnf-α stimulation according to figure legends.

### 2.5. Western Blot

For immunoblot analyses, cold RIPA buffer (ThermoFisher) was used to obtain the total protein extract from 1 × 10^6^ cells/mL. Westerns blots were performed with an equal amount of the total protein (10 μg of protein/lane for in vitro samples and 5 µg for ex vivo samples), as previously described by our group [8,9,10]. Densitometry analysis was performed using ImageLab Software v.6.1 (Bio-Rad, Hercules, CA, USA) and normalized for the loading control or total protein, as indicated in each figure legend.

### 2.6. Antibodies

The following antibodies with their respective dilutions were used for immunoblot experiments: Primary (Abcam, Cambridge, MA, USA): Total OXPHOS Rodent Cocktail (1:1000, ab110413); Interleukin-1β (1:1000, ab9722); Secondary (Abcam): Goat Anti-rabbit IgG (HRP) (1:25,000 #ab97051). Primary (Cell Signaling, Beverly, MA, USA): Drp1 (D6C7) (1:1000, #8570S); HRP Conjugate β-Tubulin (9F3) (1:1000, #5346); Secondaries (Cell Signaling): Anti-rabbit IgG (HRP) (1:3000, #7074P2), Anti-mouse IgG (HRP) (1:1000, #7076P2). Primary (Invitrogen, Thermo Fisher Scientific, Waltham, MA, USA): Opa1 (1:1000, #MA5-16149, Invitrogen); Mfn2 (1:1000, #PA5-118059, Invitrogen); Phos-Drp1 (ser616) (1:1000, # PA5-64821, Invitrogen).

### 2.7. Oxygen Consumption Rate (OCR) Measurements via Seahorse Assay

Metabolic characterization of mGFs was performed with a Seahorse XFe96 Extracellular Flux Analyzer (Seahorse Bioscience, Agilent Technology, Santa Clara, CA, USA), as previously described [8]. Briefly, 4 × 10^4^ cells were seeded into the Seahorse XF Cell Culture Microplate (Agilent Technology, Santa Clara, CA, USA) in OPTiMEM one day before the experiment, and subsequent steps were followed according to the manufacturer’s instructions, as previously described [8].

### 2.8. Mitotracker Red and CellROX Green Staining

MGFs were seeded in an 8-well imaging chamber with a coverslip bottom. After treatments, cells were stained with MitoTracker™ Red CMXRos or CellROX green. Mitotracker Red was used as a red-fluorescent dye to localize in actively respiring mitochondria in live cells. CellROX green was utilized to measure oxidative stress in live cells. Briefly, cells were stimulated with Tnf-α with or without the pretreatment with CGS21680 (1 h before). Then, cells were stained with 300 nM Mitotracker red dye or 5 µM CellROX in OptiMEM for 30 min at 37 °C, protected from light. After washing with PBS 1x, cells were fixed with a 4% formaldehyde fixative solution for 15 min at room temperature and mounted in a mounting medium containing DAPI. Images were obtained using a 63x immersion oil objective lens in a Leica Stellaris Confocal microscope (Leica Biosystems, Deer Park, IL, USA), as previously described [8].

### 2.9. Assessment of Mitochondrial Membrane Potential

Mitochondrial membrane potential was assessed with a Cell Meter™ JC-10 Mitochondrion Membrane Potential Assay Kit (AAT Bioquest, Pleasanton, CA, USA, cat#22800) following the manufacturer’s recommendations, as previously described [8]. After 24 h of treatment with or without CGS21680 (1 h before the addition of the Tnf-α stimulation), the JC-10 dye solution was added for 30 min at 37 °C in 5% CO_2_.

### 2.10. Mitochondrial DNA (mtDNA) Copy Number Quantification

The total mitochondrial and nuclear genomic DNA was collected using a PureLink Genomic DNA Mini Kit (ThermoFisher Scientific, Waltham, MA, USA), and quantified by qPCR using the Mouse Mitochondrial DNA copy number kit (Detroit R&D, Detroit, MI, USA), according to the manufacturer’s recommendations and as previously described [8].

### 2.11. Transmission Electron Microscopy Analysis

Gingival fibroblasts were fixed in 4% paraformaldehyde, 2% glutaraldehyde in 0.1 M sodium cacodylate (NaCac) buffer (pH 7.4), postfixed in 2% osmium tetroxide in NaCac, stained en bloc with 2% uranyl acetate, dehydrated with a graded ethanol series, and embedded in Epon–Araldite resin. Thin sections were cut with a diamond knife on a Leica EM UC7 ultramicrotome (Leica Microsystems, Deerfield, IL, USA), collected on copper grids, and stained with uranyl acetate and lead citrate. The sections were cut at a thickness of 80 nm. The samples were observed in a JEM 1400Flash transmission electron microscope (JEOL USA Inc., Peabody, MA, USA) at 120 kV and imaged with a OneView CCD Digital Camera (Gatan Inc., Pleasanton, CA, USA) [20]. The electron microphotographs of mitochondria were taken from each sample (*n* = 8/group) to observe mitochondrial health parameters.

### 2.12. Statistical Analysis

Statistical analysis was conducted for three independent experiments using GraphPad Prism v.10 software (GraphPad, San Diego, CA, USA) and ANOVA followed by multiple comparison tests (one-way ANOVA). Data are presented as means ± S.D.s. The cell number per well was chosen based on cell density optimization experiments for the specific assay. The significance level of *p* is indicated in each graph and in figure legends (* *p* < 0.05; ** *p* < 0.01; *** *p* < 0.001; **** *p* < 0.0001).

## 3. Results

### 3.1. Adenosine A2a Receptor Stimulation Is Protective to Alveolar Bone Loss and Mitigates Gingival Inflammation

**Figure 1:**
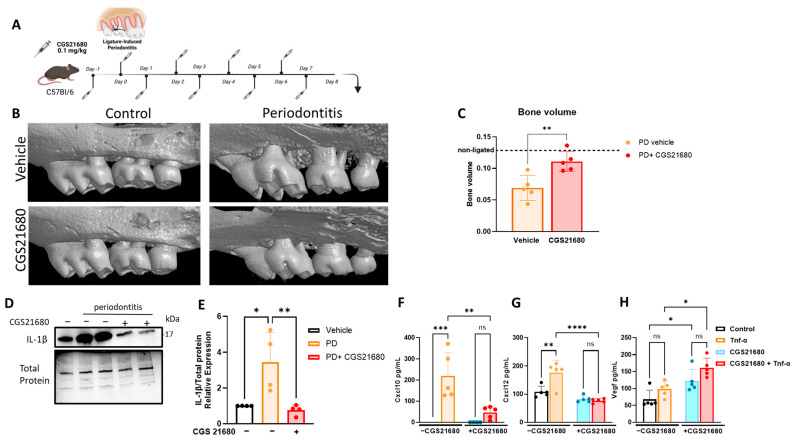
Adenosine A2a receptor (A2aR) stimulation mitigates gingival inflammation and protects from alveolar bone loss. (**A**) Ligature-induced periodontitis (LIP) mouse model with daily intraperitoneal injections of CGS21680 at 0.1 mg/Kg. (**B**) Representative 3-dimensional images and (**C**) bone volumes from hemi-maxillae of unligated control (with or without CGS21680) versus periodontitis (with or without CGS21680) from C57BL/6 mice (*n* = 5 mice/group) after 8 days. (**D**) Interleukin (IL)-1β protein expression by immunoblot of ex vivo gingival tissue extracted from LIP model with or without CGS21680 and (**E**) respective densitometry analysis relative to the total protein as a loading control. Protein levels of soluble chemokines (**F**) Cxcl10, (**G**) Cxcl12, and (**H**) vascular endothelial growth factor (Vegf) in control versus tumor necrosis factor (Tnf)-α-stimulated murine gingival fibroblasts for 24 h with or without pretreatment with CGS21680 (10 µM; 1 h before Tnf-α stimulation). Data are presented as means ± S.D.s. (* *p* < 0.05; ** *p* < 0.01; *** *p* < 0.001; **** *p* < 0.0001); ns = not significant.

Previously, we have shown the anti-inflammatory effect of extracellular adenosine in IL-1β-induced inflammatory gingival fibroblasts [8] and the protective role of adenosine-generating enzyme CD73 in alveolar bone loss [10]. This study examined whether the protective effects of adenosine in gingival inflammation and periodontitis were due to adenosine A2a receptor (A2aR) stimulation. For this, we used the adenosine receptor agonist (CGS21680) throughout the duration of our model of experimental periodontitis in vivo (Figure 1A) and as a pretreatment in vitro for Tnf-α-stimulated murine gingival fibroblasts. A2aR-agonist-treated mice showed significantly less alveolar bone loss compared to controls (vehicle-treated animals) (Figure 1B,C). Protein analysis of gingival tissue ex vivo showed ligature-induced periodontitis (LIP) induced higher pro-inflammatory IL-1β levels compared to the unligated controls and decreased IL-1β in A2aR-agonist-treated mice compared to non-treated mice (Figure 1D,E). Because gingival fibroblasts are recognized as protagonists in gingival inflammatory responses, we investigated the effects of A2aR stimulation with CGS21680 on Tnfα-induced inflammatory markers. CGS21680 significantly reduced the protein levels of inflammatory chemokines Cxcl10 and Cxcl12, while increased levels of vascular endothelial growth factor (Vegf) were detected (Figure 1F–H). These results strongly suggest that the gingival protective effects observed with adenosine in our previous studies are due to adenosine A2aR stimulation and that A2aR seems to be protective to alveolar bone, confirming the significance of the adenosinergic pathway to periodontal health.

### 3.2. A2aR Stimulation Promotes Healthy Mitochondrial Function

**Figure 2:**
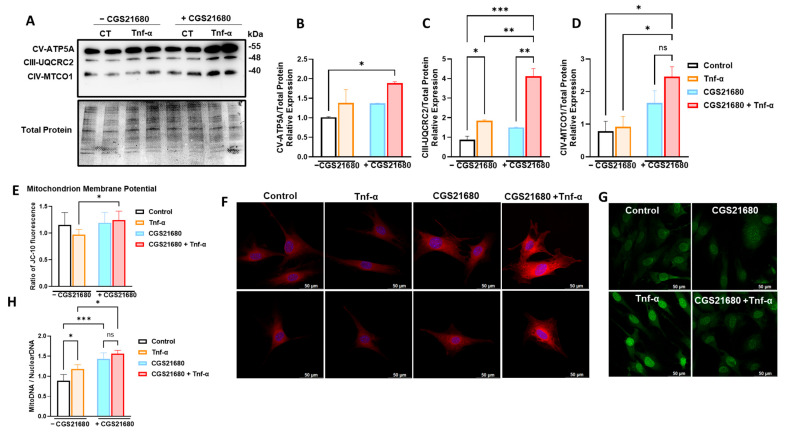
Adenosine A2a receptor (A2aR) stimulation enhances healthy mitochondrial function. (**A**) Increased mitochondrial oxidative phosphorylation (OXPHOS) detected through increased expressions of OXPHOS protein complexes in murine gingival fibroblasts (mGFs) pretreated with CGS21680 and stimulated with Tnf-α at 5 ng/mL. Densitometry analysis of subunits of (**B**) Complex V—ATP synthase subunit alpha (ATP5A), (**C**) Complex III—cytochrome b-c1 complex subunit 2 (UQCRC2), and (**D**) Complex IV—cytochrome c oxidase subunit 1 (MTCO1) relative to the total protein as a loading control. (**E**) Increased mitochondrial membrane potential in mGFs pretreated with CGS21680 with or without Tnf-α 5 at ng/mL. (**F**) Fluorescence microscopy of Mitotracker red staining and (**G**) oxidative stress detected through CellROX green staining in mGFs with or without CGS21680 at 10µM and stimulated with Tnf-α at 5 ng/mL. (**H**) Relative quantification of mitochondrial-DNA-to-nuclear-DNA load by quantitative real-time Polymerase Chain Reaction (PCR). Data are presented as means ± S.D.s. (* *p* < 0.05; ** *p* < 0.01; *** *p* < 0.001); ns = not significant.

Mechanistically, we and others have shown extracellular adenosine promotes mitochondrial biogenesis and healthier mitochondrial function in fibroblasts [8,21] but without a clear understanding of the specific adenosine receptor involved. To this end, we included multiple readouts related to mitochondrial health in the presence of the selective adenosine receptor agonist (CGS21680) with or without Tnf-α stimulation, simulating an inflammatory microenvironment in vitro. We first investigated the abundance of electrontransport chain (ETC) proteins involved in oxidative phosphorylation, and we showed that ETC Complexes III, IV, and V were significantly upregulated in the presence of A2aR stimulation (Figure 2A–D). Because the detection of increased levels of mitochondrial complexes does not really convey information about mitochondrial function, we have also checked the membrane potential. We have detected higher mitochondrial membrane potential in the presence of Tnf-α stimulation with CGS21680 (Figure 2E) in addition to higher mitochondrial mass via mitotracker red staining (Figure 2F) and lower reactive oxygen species levels (Figure 2G) with the addition of CGS21680 prior to Tnf-α stimulation, suggesting a significant effect on healthy mitochondrial function in the presence of A2aR agonism. We have also demonstrated higher loads of mitochondrial DNA relative to nuclear DNA when cells were pretreated with CGS21680 with or without Tnf-α stimulation (Figure 2H). Collectively, these data suggest healthier mitochondrial function in the presence of A2aR stimulation.

### 3.3. A2aR Stimulation Leads to Decreased Tnf-α-Induced Mitochondrial Stress and Elongated Mitochondria

**Figure 3:**
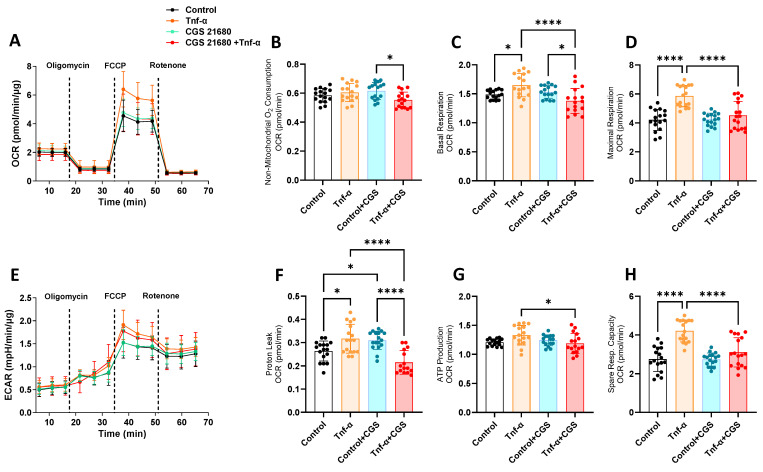
Adenosine A2aR stimulation leads to decreased Tnf-α-induced mitochondrial stress and proton leak. (**A**–**D**) Oxygen consumption rate (OCR) of mGFs during Seahorse Mito Stress Test in mGFs pretreated with CGS21680 (10 µM) and stimulated with Tnf-α at 5 ng/mL for 6 h. (**E**–**H**) Extracellular acidification rate (ECAR) and OCR parameters of mitochondrial function, comparing experimental groups. Data are presented as means ± S.D.s. (* *p* < 0.05; **** *p* < 0.0001), and dots represent each technical replicate. Abbreviations: Carbonyl cyanide p-trifluoro-methoxyphenyl hydrazone (FCCP). Adenosine triphosphate (ATP).

To complement our investigation into the mitochondrial function, our next step was to monitor the oxygen consumption rate (OCR) through a real-time metabolic assay (Seahorse analysis) in the presence or absence of adenosine agonist CGS21680 with or without Tnf-α through the mitochondrial stress test. Tnf-α stimulation resulted in significantly higher levels for most of the OCR parameters measured through the Seahorse metabolic assay (Figure 3A–H). These data show higher levels of mitochondrial stress under Tnf-α stimulation (such as proton leak, spare respiratory capacity, and maximal respiration), which were significantly dampened by CGS21680, confirming the mitoprotective effect of adenosine receptor A2aR stimulation on inflammatory-induced fibroblasts.

**Figure 4:**
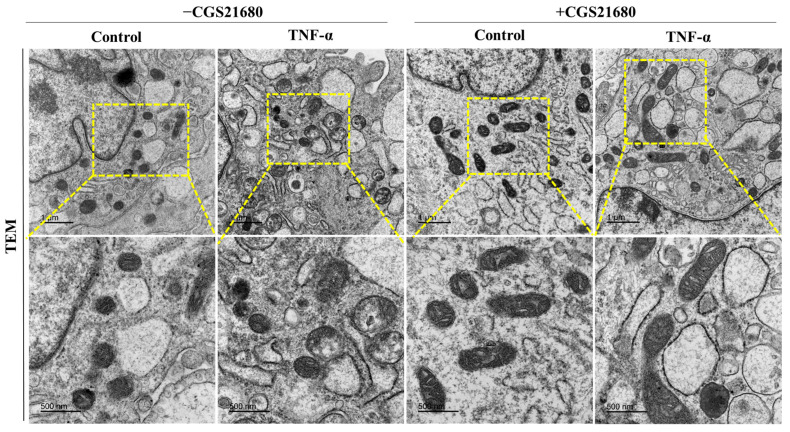
Adenosine A2aR stimulation leads to more elongated healthy mitochondria. Representative electronic micrographs featuring the mitochondria ultrastructure (scale bar = 1 µm, (**upper panel**)) in murine gingival fibroblasts pretreated with CGS21680 (10 µM) and stimulated with Tnf-α at 5 ng/mL, with the corresponding high-magnification insets (yellow-dashed area—scale bar = 500 nm, (**lower panel**)) for each group.

To investigate the mitochondrial morphology and any possible changes in the mitochondrial ultrastructure, we performed a transmission electron microscopy (TEM) examination of the experimental groups (Figure 4). In the absence of CGS21680, the group of cells treated with Tnf-α exhibited alterations of the shape and size of the mitochondria, with an aspect of swelling organelles with potential fragmentation. The yellow-dashed squares show areas of interest, with higher magnification shown in the lower panels. Cells treated with CGS21680 demonstrated more elongated mitochondria with more prominent electron density, strongly suggesting healthier mitochondrial dynamics and quality control.

### 3.4. Mitoprotective Effect of A2aR Stimulation Is Due to Improved Mitochondrial Dynamics Through Increased Mitochondrial Fusion

**Figure 5:**
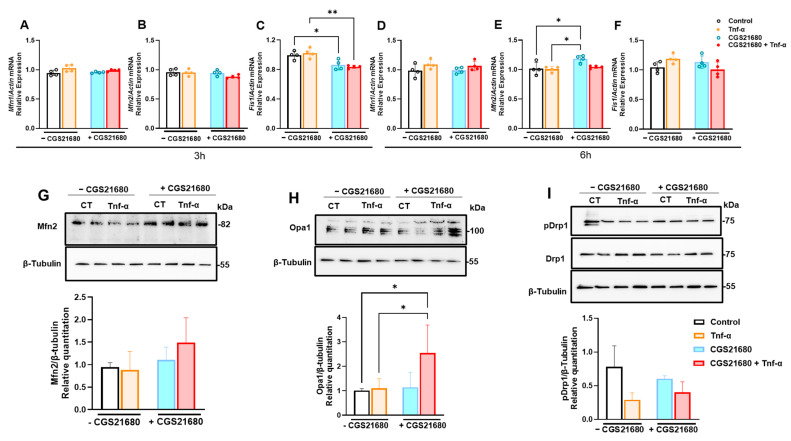
Mitoprotective effect of adenosine A2aR stimulation is due to improved mitochondrial quality control through increased mitochondrial fusion. (**A**–**F**) MRNA levels of Mitofusins 1 and 2 (*Mfn1/2*) and Fission 1 (*Fis1*) in murine gingival fibroblasts pretreated with CGS21680 (10 µM) and stimulated with Tnf-α at 5 ng/mL for (**A**–**C**) 3 h or (**D**–**F**) 6 h. Immunoblots showing protein expressions of mitochondrial quality control targets pro-fusion (**G**) Mfn2 and (**H**) Opa1 and pro-fission (**I**) pDrp1 and Drp1, with their respective densitometry analysis relative to β-Tubulin as a loading control. Data are presented as means ± S.D.s. (* *p* < 0.05; ** *p* < 0.01).

Given the differences observed in the mitochondrial morphology, we proceeded with an investigation of the main proteins involved in mitochondrial dynamics, such as regulators of mitochondrial fusion (mitofusins 1 and 2—Mfn1/2), Opa1, and mitochondrial fission (Fis-1 and Drp-1), as observed in Figure 5. Fusion and fission cycles are controlled by a group of mitochondrial proteins that are central to every aspect of mitochondrial dynamics, as reviewed elsewhere [22]. At the mRNA expression level, we checked Mfn1/2 and Fis1 (Figure 5A–F). We detected lower Fis-1 mRNA levels at an early time point (after 3 h; Figure 5C) and higher Mfn-2 levels later (after 6 h; Figure 5E). Although not statistically significant, protein levels of Mfn2 showed a trend for increasing with the addition of CGS21680 (Figure 5G), and Opa1 levels were significantly higher in the presence of CGS21680 compared to control or Tnf-α-stimulated cells (Figure 5H), confirming the elongated aspect observed in EM with CGS21680 (Figure 4), was probably indicative of increased mitochondrial fusion in mGFs. The phosphorylation of Drp-1, which is highly indicative of mitochondrial fission, remained unchanged with CGS21680 (Figure 5I), suggesting the mitoprotective effects seen in the previous readouts were most likely due to the promotion of mitochondrial fusion rather than any changes in fission improving mitochondrial dynamics in the presence of A2aR stimulation.

## 4. Discussion

In the present study, our main finding was that adenosine A2a receptor stimulation mitigated periodontitis. At the cellular level, we show for the first time that the mitoprotective effects of adenosinergic signaling via A2aR in gingival fibroblasts occurred by enhancing mitochondrial dynamics in favor of increased mitochondrial fusion.

Our in vivo findings that CGS21680-treated mice exhibited reduced alveolar bone loss and lower gingival IL-1β levels corroborate previous studies showing that A2aR-specific agonists protect long bones in inflammatory contexts by inhibiting osteoclastogenesis in vivo and in vitro by inhibiting M-CSF–RANKL-stimulated osteoclast differentiation and function [23]. Mechanistically, this mirrors previous findings from our group, where we showed the protective effects of the adenosine-generating enzyme (CD73) in preventing bone loss (in long bones and in alveolar bone with experimental periodontitis) via osteoclast energy metabolism and by regulating the hyper-inflammatory response of gingival fibroblasts.

In osteoblast precursors, existing literature shows A2aR activation altered cytoskeleton signaling, RANKL, and OPG secretion and promoted Wnt signaling to promote osteoblast differentiation and inhibit osteoclast differentiation [24]. Together, these data strongly reinforce that adenosinergic agonism via A2aR is a central mediator of adenosine’s periodontal-bone protective and anti-inflammatory effects. A previous study using another ligand of A2aR, known as polydeoxyribonucleotide (PDRN), in an experimental periodontitis model in rats highlighted the role of the A2a receptor in resolving gingival inflammation and suggested adenosine A2aR stimulation might be an innovative strategy for the treatment of periodontitis [25]. In fact, PDRN has well-known tissue-damage preventive properties mediated by A2aR and has been considered as a promising skin anti-aging agent due to its biological activity promoting mitochondrial biogenesis [26]. Adenosine signaling via A2aR also enhanced IL-10 production in macrophages, which was associated with increases in mitochondrial fusion and membrane potential, indicating improvement in mitochondrial health [27].

Our observations of the elevated abundance of ETC complex proteins, higher membrane potential, mitochondrial mass, mtDNA content, and reduced ROS in CGS21680-treated cells support our hypothesis that A2aR signaling enhances mitochondrial fitness. Prior work on osteoarthritis chondrocytes from human and mice shows A2aR agonism promotes mitochondrial dynamics, reduces oxidative injury, and supports autophagy/mitophagy [17]. The fibroblast results demonstrated in the present study extend this to gingival inflammation, indicating that A2aR stimulation through CGS21680 can directly counteract Tnf-α-induced mitochondrial dysfunction, in line with research showing improved chondrocyte survival and mitochondrial dynamics via A2aR agonists. Previous reports by our group showed the direct effect of adenosine stimulation on dampening the hyper-inflammatory state of human gingival fibroblasts through their metabolic reprogramming toward mitochondrial biogenesis [8]. We have also previously demonstrated that adenosine receptor activation leads to increases in heme-oxygenase 1 (HO-1), which is a stress-responsive enzyme important for defense against oxidant-induced injury, and phosphorylated-adenosine-monophosphate-activated protein kinase (pAMPK), a key regulator of cellular metabolism [9].

In our interpretation, the Seahorse data showing that CGS21680 decreases proton leak, maximal respiration, and spare capacity under Tnf-α stress confirms that adenosinergic activation buffers mitochondrial overwork and reduces cell stress. This functional protection of mitochondria complements morphological findings via TEM, where CGS21680 prevents mitochondrial swelling and fragmentation while promoting mitochondrial elongation, consistent with enhanced mitochondrial fusion and quality control in our model. These results suggest that the A2aR-mediated maintenance of the mitochondrial ultrastructure is important to preserve cellular homeostasis under inflammatory conditions. The shift toward fusion-mediated dynamics, reflected by the upregulation of well-characterized mitochondrial fusion regulators (such as Opa1 and Mfn2) [28,29] and downregulation of the fission marker Fis-1 [30] after CGS21680 treatment, suggests A2aR activation supports mitochondrial integrity and limits ROS generation under mitochondrial stress. High levels of ROS, if not counterbalanced by an efficient antioxidant system, promote mitochondrial fragmentation, swelling, or shortening, whereas a reduction in ROS leads to mitochondrial filamentation (or elongation), as reviewed elsewhere [31]. Notably, considering the limitations of our study, which cannot be extrapolated for other cell types for in vitro data and do not represent long-term data for the experimental periodontitis model, our findings complement existing literature on the cell protective effects of adenosinergic stimulation via A2aR and offer a valuable mechanistic insight missing from earlier studies. Future directions include providing deeper mechanistic understanding and translational relevance, potentially pointing toward the therapeutic modulation of A_2_aR in inflammatory diseases.

## 5. Conclusions

In conclusion, A2aR stimulation attenuates alveolar bone loss and gingival inflammation, exerting a mitoprotective effect on gingival fibroblast bioenergetics and reducing oxidative stress. Adenosinergic stimulation enhances mitochondrial fitness and morphology, ensuring periodontal integrity. Together, our data emphasize mitochondrial dynamics as a central mediator—specifically through fusion-promoting pathways—as a novel axis in A2aR-driven cytoprotection and cellular resilience. This expands upon existing evidence and identifies mitochondrial maintenance as an underappreciated prevention/therapeutic target in periodontitis and related bone-resorptive diseases.

## Data Availability

All the data generated and/or analyzed during the current study are included in this published article.
